# FLT3-TKD Measurable Residual Disease Detection Using Droplet Digital PCR and Clinical Applications in Acute Myeloid Leukemia

**DOI:** 10.3390/ijms25115771

**Published:** 2024-05-26

**Authors:** Eric Wenlong Li, Ngoc Yen Kim Tran, Derek McCulloch, Michael Krigstein, Alberto Catalano, Jad Othman, Edward Abadir, Cheryl Smith, Harry Iland

**Affiliations:** 1 Institute of Hematology, Royal Prince Alfred Hospital, Sydney, NSW 2050, Australia; 2 Molecular Hematology Laboratory, Royal Prince Alfred Hospital, Sydney, NSW 2050, Australia; 3 Faculty of Medicine and Health, The University of Sydney, Sydney, NSW 2050, Australia; 4 Department of Hematology, St Vincent’s Hospital, Sydney, NSW 2010, Australia; 5 Department of Hematology, Royal North Shore Hospital, Sydney, NSW 2065, Australia

**Keywords:** acute myeloid leukemia, clonal evolution, treatment monitoring, FMS-like tyrosine kinase, measurable residual disease, precision oncology

## Abstract

The tyrosine kinase domain of the FMS-Like tyrosine kinase 3 (*FLT3*-TKD) is recurrently mutated in acute myeloid leukemia (AML). Common molecular techniques used in its detection include PCR and capillary electrophoresis, Sanger sequencing and next-generation sequencing with recognized sensitivity limitations. This study aims to validate the use of droplet digital PCR (ddPCR) in the detection of measurable residual disease (MRD) involving the common *FLT3*-TKD mutations (D835Y, D835H, D835V, D835E). Twenty-two diagnostic samples, six donor controls, and a commercial D835Y positive control were tested using a commercial Bio-rad^®^ ddPCR assay. All known variants were identified, and no false positives were detected in the wild-type control (100% specificity and sensitivity). The assays achieved a limit of detection suitable for MRD testing at 0.01% variant allelic fraction. Serial samples from seven intensively-treated patients with *FLT3*-TKD variants at diagnosis were tested. Five patients demonstrated clearance of *FLT3*-TKD clones, but two patients had *FLT3*-TKD persistence in the context of primary refractory disease. In conclusion, ddPCR is suitable for the detection and quantification of *FLT3*-TKD mutations in the MRD setting; however, the clinical significance and optimal management of MRD positivity require further exploration.

## 1. Introduction

Acute myeloid leukemia (AML) is a hematological malignancy characterized by the proliferation and impaired differentiation of myeloid blast cells that expand in the bone marrow and peripheral blood [1]. A suspected diagnosis of AML is confirmed by hematological and morphological assessment, immunophenotyping, cytogenetic analysis, and molecular profiling for structural aberrations and genetic mutations. Detecting AML genetic markers is essential for risk stratification, refining treatment options, and optimizing disease management [1,2].

FMS-like tyrosine kinase 3 (*FLT3*) is a transmembrane receptor that has a significant role in controlling cell proliferation, survival, and differentiation through various signaling transduction pathways [3]. Thirty percent of AML patients have an *FLT3* mutation. The most common are *FLT3* internal tandem duplications (*FLT3*-ITD) in the juxtamembrane domain (~25%) and missense mutations in the activation loop of the tyrosine kinase domain (*FLT3*-TKD) (5–10%). These mutations are highly expressed in leukemic cells and constitutively active, thus contributing to leukemogenesis. The 2022 ELN guidelines state that *FLT3*-ITD with or without *NPM1* is associated with intermediate risk, whereas *FLT3*-TKD has no demonstratable prognostic impact [4,5]. Nevertheless, detection is important for effective clinical decision making, such as administering FLT3-targeted drug therapy (e.g., midostaurin and gilteritinib) [6,7]. Monitoring measurable residual disease (MRD) is also important to detect molecular persistence, drug resistance and risk of relapse [8].

There is currently no standard method of *FLT3*-TKD monitoring. A frequently used method for the initial detection of variants is multiplex Polymerase Chain Reaction (PCR) followed by restriction digestion and capillary electrophoresis (CE), which simultaneously detect *FLT3*-ITD and *FLT3*-TKD mutations at D835 and I836 [9,10]. Although this technique has a rapid turnaround time, the sensitivity is limited at ~5%, thus preventing its use in MRD detection [3]. Locally at our center, the High-Resolution Melt (HRM) assay is used for *FLT3*-TKD variant detection, followed by Sanger sequencing, which suffers from a similar sensitivity limit of 5–20%.

Increasingly, droplet digital PCR (ddPCR) has been used as a highly sensitive technique in detecting variants with the ability to provide absolute quantification and superior precision compared to other methods. In this article, we report on the performance of an optimized protocol for detecting the most common *FLT3*-TKD variants (D835Y, D835V, D835H, and D835E) using a commercial Bio-Rad ddPCR system. Furthermore, we describe the clonal trajectories of *FLT3*-TKD variants following treatments as a proof of concept.

## 2. Results

### 2.1. Accuracy

We performed ddPCR testing on diagnostic samples previously analyzed using a validated method (Table 1). These include HRM, Sanger sequencing, NGS, and CE. There were 12 D835Y, 5 D835H, 4 D835V and 1 D835E diagnostic samples. Using the appropriate assay, ddPCR detected all previously known variants. Furthermore, DNA extracted from peripheral blood samples from six healthy donors were also tested using each assay without false positives. This results in a specificity and sensitivity of 100%.

### 2.2. Limit of Detection (LoD)

The limit of detection (LoD) is defined as the lowest concentration/analyte detected with 95% confidence. The LoD depends on the input DNA and the level of false positive droplets (which, in turn, is dependent on the positive/negative threshold setting). The LoD was tested using 132 ng of DNA input per well to achieve a theoretical LoD of 0.01% [11]. The amplitude threshold for positive droplets was set at 3500 for the D835Y and V assays, 4000 for the D835E assay, and 4500 for the D835H assay for maximum specificity and sensitivity. Each sample was tested in triplicate, and three positive droplets over the three replicates were required for a positive call. As up to 16 replicates were performed in the dilution studies, the positivity rate is calculated based on the total triplicate combinations of all replicates. As shown in Table 2, the lowest allelic fraction detected in all four assays was approximately 0.01%.

### 2.3. Limit of Blank (LoB)

The limit of blank is based on the number of false positive droplets when wild-type samples are tested. Each assay was used on six healthy donor peripheral blood samples, tested in duplicates with DNA input of 132 ng and sample amplitude thresholds as LoD studies. No false positive droplets were detected using the D835Y assay. The D835V assay recorded two droplets over 12 replicates (resulting in no false positive calls). The D835E assay detected 1 positive droplet over 12 replicates (no false positive calls), and the D835H assay detected 2 positive droplets over 11 replicates (no false positive calls).

### 2.4. Linearity

**Figure 1:**
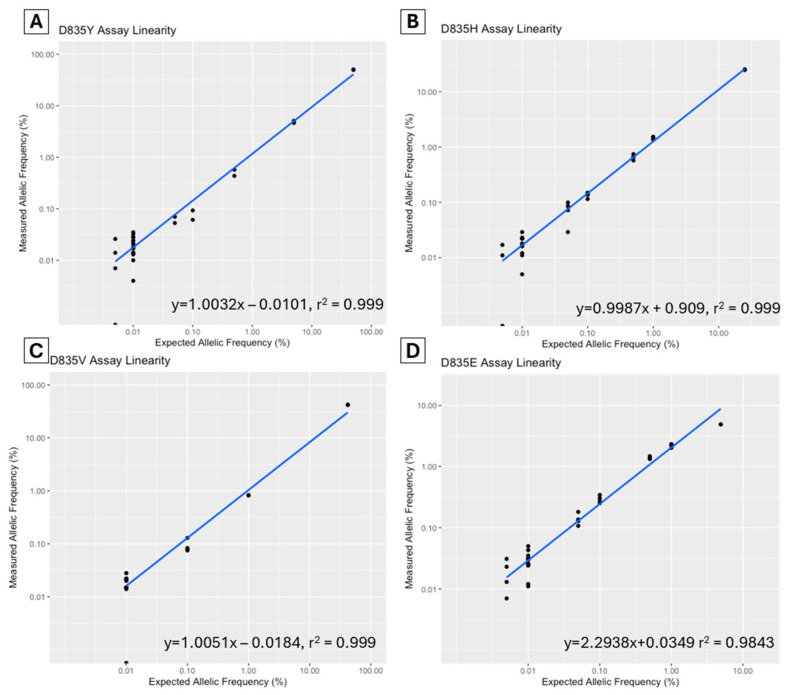
Assay linearity of *FLT3*-TKD D835Y (**A**), D835H (**B**), D835V (**C**) and D835E (**D**) assays in dilutional studies using ddPCR (Bio-Rad, Hercules, CA, USA).

A dilution study was performed to assess the linearity of each assay. The allelic fraction was plotted to determine the correlation coefficient (R^2^) (Figure 1). For the D835Y assay, a commercial control (Horizon Discovery Ltd., Cambridge, UK, Catalog ID HD668) was serially diluted to a range of 50–0.005% allelic frequency. The D835V, D835H, and D835E diagnostic samples (sample ID V2, H5, and E1) were serially diluted from various diagnostic levels to 0.005%. The R^2^ for all assays was >0.999 except for the D835E assay, which was 0.984.

### 2.5. Precision

The intra-run variability was assessed for the D835Y assay using a commercial control, while samples with known variants were used for the D835V/H/E assays (Table 3). Variability was tested at 1%, 0.1%, and 0.01%. The coefficient of variation (CV) was 1.1–6.6% at 1% allelic frequency with higher CV noted at the limit of detection of 0.01% allelic frequency (CV range 38.9–54.2%).

A limited inter-run variability study was performed using diagnostic patient samples over multiple runs on different days, and all assays demonstrated a CV of 1% or less (at diagnostic allelic frequency, Table 4).

### 2.6. Robustness

The assay performance was tested by varying the PCR annealing temperature and digestion enzyme. A temperature gradient of 53–58 °C was assessed, and the *Mse*I (Takara Bio^®^, San Jose, CA, USA, Cat no. 1247A) digestion enzyme was used in addition to the manufacturer-recommended enzyme *Hae*III. This showed the optimal annealing temperature of 53–56.1 °C (final protocol temperature 55 °C), which maintained a satisfactory separation of positive and negative clusters, and it was associated with consistent results (median CV% for VAF over the optimal temperature range of 1.6%, range 1.0–4.5%). There was no appreciable effect of digestion enzyme change (Supplementary Figure S1).

### 2.7. Assay Cross-Reactivity

**Figure 2:**
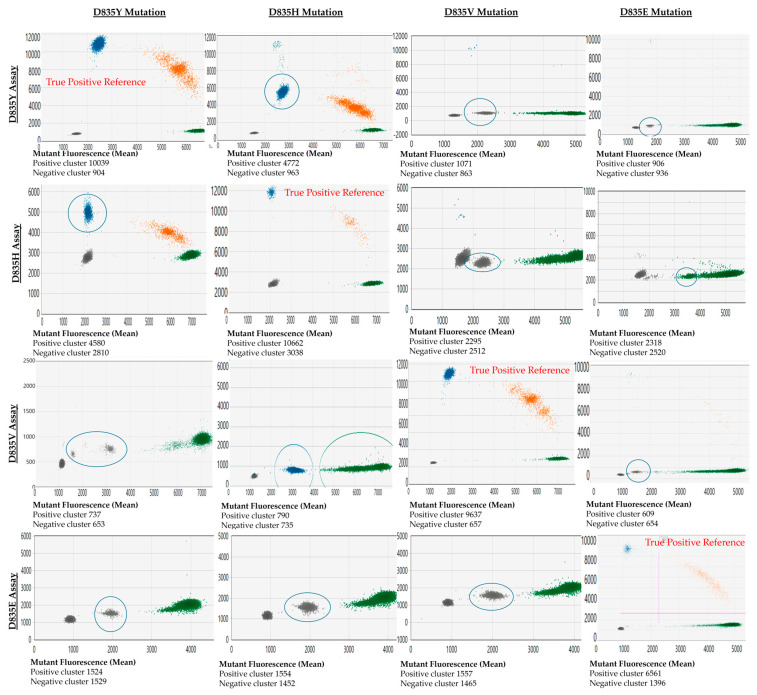
Demonstration of cross-reactivity between D835 assays and each common D835 mutation. Output from QX Manager Standard Edition version 1.2 (Bio-Rad, Hercules, CA, USA). The *x*-axis represents the fluorescence of the wild-type channel (HEX dye), and the *y*-axis represents the fluorescence from the mutant channel (FAM dye). The cross-reactive clones are highlighted with a blue circle. Blue clusters correspond to droplets designated by the user or the software to have mutant allele only, green clusters are designated wild-type allele only, and orange cluster has both mutant and wild-type allele.

The cross-reactivity between *FLT3*-TKD assays against other variants they were not specific for (off-target variants) was noted. The off-target variants may present as droplet clusters with a significantly decreased fluorescence amplitude in that mutant channel compared to the true positive variant (e.g., testing D835H variants with a D835Y assay). Alternatively, the mutant clones may present as distinct clusters in the wild-type channel (e.g., D835E assay with D835Y, D835H, and D835V variants). The appearance of these cross-reactive droplets and their associated mutant fluorescence amplitude relative to the actual positive events (reference) is shown in Figure 2.

### 2.8. Clinical Utility

**Figure 3:**
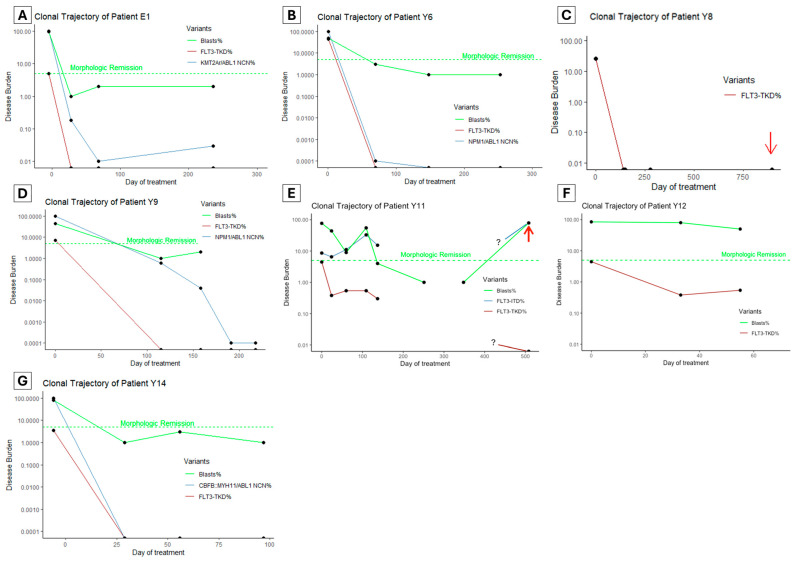
(**A**–**G**) The clonal trajectory of patients with known *FLT3*-TKD undergoing intensive chemotherapy. Red arrows indicate when the patient experienced frank hematologic (**E**) or soft tissue relapse (**C**). The green dashed line indicates a 5% blasts threshold below which a morphologic remission is defined. Samples were unavailable for an extended duration for patient Y11 (**E**), and the clonal trajectories during this time are unknown (as denoted by the ? symbol in the graph). NCN: normalized copy number, where NCN% is the oncogenic transcript copy number to ABL1 copy number ratio relative to the ratio at diagnosis.

To assess the clinical utility of *FLT3*-TKD monitoring in clinical practice, we retrospectively performed *FLT3*-TKD MRD testing using ddPCR on patients between 1 January 2017 and 1 January 2024 who had AML and *FLT3*-TKD mutations with at least three serial samples available. Seven patients were identified (Figure 3A–G, numeric results in Supplementary Table S1), of whom all had ‘7 + 3’ intensive chemotherapy (cytarabine and anthracycline) with midostaurin as the first induction treatment.

Five patients (Figure 3A–D,G) achieved undetectable *FLT3*-TKD clones at first post-treatment MRD testing (28–115 days since treatment initiation). Among these, four patients had an alternative marker, and two (Patient Y6 and Y14, Figure 3B,G) achieved an optimal response per ELN guidelines (based on *NPM1* and *CBFB::MYH11* monitoring). In the other two patients, one had *KMT2A::ELL* rearrangement with persistent detectable disease (LoD VAF 0.01%), which increased to 0.03% by the end of consolidation and proceeded to allogeneic hematopoietic cell transplant (alloHCT). The second patient Y9 (*NPM1*, *SF3B1*, *NRAS*, *FLT3*-TKD and *FLT3*-ITD variants at diagnosis) achieved a >3 log reduction in *NPM1* after two cycles of chemotherapy (*FLT3*-ITD not monitored); then, it had a persistent *NPM1* clone below quantifiable levels (<10 copies) by the end of consolidation (Figure 3D). This patient proceeded to alloHCT but suffered a fatal hemorrhagic complication.

Patient Y8 (Figure 3C) had *KMT2A::MLLT4* rearrangement, which was not monitored using MRD, but had rapid clearance of the *FLT3*-TKD clone after the first induction. This patient experienced isolated soft tissue (chloroma) relapse over one year after alloHCT, with undetectable *FLT3*-TKD and *KMT2A* rearrangement in the bone marrow, but no material available for testing from the soft tissue.

Of the two patients with persistent *FLT3*-TKD, the first (patient Y11, Figure 3E) had a complex genomic profile at diagnosis (*FLT3*-ITD, *KRAS*, *ASXL1*, *SF3B1* and *RUNX1*). Initial 7 + 3 induction, followed by FLAG-Ida and venetoclax–azacitidine, all failed to induce a morphologic remission. Instead, there was an increase in the *FLT3*-ITD clone (from VAF 8.63% to 32.95%) and a persistent low level *FLT3*-TKD clone only detectable using ddPCR. Interestingly, the second generation FLT3 inhibitor gilteritinib was initiated, which resulted in morphologic remission and a modest reduction in *FLT3* variant clones (*FLT3*-ITD 32.95% reduced to 15.34%, *FLT3*-TKD 0.54% reduced to 0.3%). This patient subsequently underwent alloHCT but experienced hematologic relapse 11 months later associated with high *FLT3*-ITD burden but undetectable *FLT3*-TKD burden. The other patient (patient Y12, Figure 3F) had mutations in *PHF6*, three distinct clones of *NRAS*, and *FLT3*-TKD. Initial induction failed to induce morphologic remission; then, FLAG-Ida with venetoclax followed by Venetoclax and Azacitidine achieved a morphologic remission (subsequent loss of follow-up without MRD data).

## 3. Discussion

Historically, the assessment of disease response in AML is based on a microscopic examination of bone marrow blast count with the negative threshold set at 5%. At this level, there still may be over a million residual leukemic cells that drive disease relapse in patients deemed in ‘complete’ remission [12]. Therefore, there is a need to have available sensitive assays to detect MRD and allow the early detection of disease recurrence to guide therapeutic intervention such as targeted therapies or stem cell transplantation. The European LeukemiaNet (ELN) has established specific guidelines for the evaluation of MRD and outlines the use of multiparameter flow cytometry (MFC-MRD) and molecular methods (including qPCR and dPCR). The ELN also stipulates suitable molecular biomarkers, highlighting leukemia initiating variants (e.g., *NPM1*, *CBFB::MYH11*, *RUNX1::RUNX1T1*, *PML::RARA*) essential in disease monitoring [8]. In contrast, germline variants and those associated with clonal hematopoiesis (e.g., *ASXL1*, *TET2* and *DNMT3A*) are not recommended for disease monitoring. *FLT3* mutated clones (ITD and TKD) are considered sub-clonal, where their detection likely reflects residual disease, but their absence does not exclude disease relapse [8]. Furthermore, *FLT3* variants at diagnosis can be lost during the disease course with novel *FLT3* variants found during disease relapse [13].

Currently, there is no standard method for the MRD monitoring of *FLT3* variants. Recently, advances in NGS-based methodologies have significantly improved sensitivity and capability for MRD testing. Firstly, a targeted error-corrected NGS panel that covers both *FLT3*-ITD and TKD has achieved a limit of detection of 0.01% [14]. Using this method, it has been shown in the Pre-MEASURE study that detectable *FLT3*-ITD before alloHCT was associated with a significantly higher risk of relapse (3-year risk of 26% for *FLT3*-ITD^−^ vs. 67% in those *FLT3*-ITD^+^, *p* < 0.001), and inferior survival (3-year overall survival, 63% for *FLT3*-ITD^−^ vs. 31% for *FLT3*-ITD^+^) [14]. In addition, early data (abstract form) suggests the persistence of *FLT3*-TKD variants (VAF ≥ 0.1% but not 0.1–0.01%) is strongly associated with the risk of disease relapse (73.8% vs. 20.5%, *p* < 0.0001) and reduced survival after transplant (11.4% vs. 66.8%, *p* < 0.001) compared to those testing negative [15].

However, the above commercially available NGS method is labor-intensive and potentially cost-prohibitive for many diagnostic laboratories, particularly when testing is required at numerous time points. Less costly NGS options include open-source bioinformatic pipelines such as getITD [16], which was developed for monitoring *FLT3*-ITD and similarly demonstrated prognostic significance in the alloHCT setting [17]. Most recently, preliminary data have revealed the development of an automated system (Cepheid GeneXpert^®^) for both *FLT3*-ITD and *FLT3*-TKD with a sensitivity of approximately 0.01% [18,19].

In this context, the results from this validation study demonstrate that the ddPCR assay is a robust methodology that is simple, cost-effective and labor-efficient and achieves a limit of detection parallel to advanced NGS techniques with a faster turn-around time. The commercially available targets assessed in this study (D835Y, D835V, D835H, and D835E) cover a significant proportion of patients with *FLT3*-TKD mutations. Furthermore, reproducibility studies support the excellent precision of ddPCR assays. However, the variability is higher at the LoD of 0.01% (CV ~50%), but this is satisfactory for clinical use, considering the ELN definition of molecular relapse is ≥1 log increase [2].

A limitation of this validation study is that less common *FLT3*-TKD mutations (e.g., D835A, and I836del) were not included. Whilst ddPCR assays may be available commercially, the performance of these assays needs to be further assessed. Furthermore, targeted ddPCR assays for variants at non-canonical *FLT3*-TKD sites may not be available commercially. NGS methods mentioned previously may be required to monitor these variants sensitively (if deemed pathogenic) [20]. A second limitation of the assay is that there is a cross-reactivity of assays with related mutations (e.g., the D835Y assay detects D835V/H/E/I836del variants). However, this study showed that the fluorescence amplitude of the cross-reactive variant is clearly distinguishable from the target variant’s amplitude. The knowledge of this cross-reactivity is essential in *FLT3* monitoring given that new variants may arise with disease relapse. For this reason, we provided a cross-reactivity map diagnosticians can refer to for this assay (Figure 2).

An important consideration when implementing this assay into clinical practice is determining the clinical significance of *FLT3*-TKD MRD. As discussed, there is increasing evidence of the adverse prognostic impact of *FLT3*-ITD and TKD residual disease in patients undergoing an alloHCT [14,15,17]. For *FLT3*-TKD, the question that remains from the Pre-MEASURE study is whether <0.1% MRD positivity is associated with adverse clinical outcomes [15]. Furthermore, it is uncertain whether *FLT3*-TKD provides additional prognostic information if co-occurring with other established biomarkers.

Another pertinent question is whether intervention (and which treatment) at the time of *FLT3*-TKD molecular relapse can improve patient outcomes. A recent retrospective study found that initiating a FLT3-inhibitor during molecular relapse (based on a non-*FLT3* MRD marker, e.g., *NPM1*) effectively induced MRD negativity with patients successfully taken to salvage alloHCT [21]. However, the number of patients with *FLT3*-TKD was low (n = 10), and the *FLT3*-TKD disease burden at relapse is unknown. Prospective clinical trials investigating the efficacy and safety of early MRD-guided therapy, such as the ongoing Australian INTERCEPT trial [22], will be valuable in determining the optimal treatment strategy in molecular relapse.

## 4. Materials and Methods

### 4.1. Sample Preparation

White blood cell pellets were isolated from peripheral blood and bone marrow collected in EDTA. DNA extraction was performed using a Maxwell RSC (Promega, Fitchburg, WI, USA) extractor and Maxwell RSC Buffy Coat DNA Kit (Promega, Fitchburg, WI, USA) as per manufacturer instructions. DNA concentration and quality/purity were determined by Nanodrop 2000 Spectrophotometer (Thermoscientific, Waltham, MA, USA) and stored at −30 °C before testing. On the day of testing, diagnostic and MRD DNA samples were diluted to 10 ng/µL and 20 ng/µL, respectively.

### 4.2. ddPCR Method

The PCR mix for each reaction is comprised of 11 µL of ddPCR Supermix for Probes (no dUTPs) x2 concentration (Bio-Rad, California, USA), 1.1 µL of PrimePCR ddPCR Mutation Detection Assay Kit (D835Y, D835H, D835V, D835E) (Bio-Rad, California, USA assay ID dHsaMDV2010047, dHsaMDV2510492, dHsaMDV2516838, dHsaMDV2516864), 0.4 µL of *Hae*III Restriction Enzyme (10,000 units/mL) (NEbiolabs, Ipswich, MA, USA), 2.9 µL of nuclease-free water (Ambion, Waltham, MA, USA) and 6.6 µL of gDNA (132 ng/well for MRD samples and 66 ng/well for diagnostic samples). A no-template control (NTC), negative control and positive control were tested with every experiment. Elution Buffer (Promega, Fitchburg, WI, USA) was used as NTC. Negative controls were samples from healthy donors previously tested as negative for *FLT3*-TKD by orthogonal methods (Table 1). Positive controls include the commercially available *FLT3*-TKD D835Y reference standard (Horizon Discovery Ltd., Cambridge, UK), and for D835H, D835V, and D835E assays, samples from patients with known *FLT3*-TKD mutations were used, as no commercial controls were available.

Droplet generation was performed using the QX200 Droplet Generator (Bio-Rad, California, USA) as per manufacturer instructions. The thermocycling conditions include 95 °C for 10 min (1 cycle), 94 °C for 30 s (40 cycles), 55 °C for 1 min (40 cycles), and 98 °C for 10 min (1 cycle). The Ramp rate is 2 °C/s. The droplets were read using the QX200 Droplet Reader (Bio-Rad, CA, USA). A negative, positive (mutant assay specific) and no template control were run in each assay to check for result validity. Positive vs. negative thresholds were identified manually by finding the midpoint between the average positive and negative droplet clusters, and the threshold was applied across all samples with the same primers during each run. The allelic fraction is reported as ‘Fractional abundance’ by the Bio-Rad software QX Manager version 1.2 (California, USA), which is calculated using the following formula:

F (%) = A/(A + B) × 100(1)

Equation (1) was used to calculate the average fractional abundance of the target gene concentration (A) relative to the reference gene concentration (B).

### 4.3. Performance Assessment and Statistics

Accuracy: Accuracy is assessed by comparing results from the comparator test (ddPCR in this case) against an accepted and accredited reference standard. The reference standards used in this study include results from two NGS panels where available (Haematological Malignancy (ALLHAEM) Gene Panel using QIAGEN QIAseq single primer extension-based chemistry (QIAGEN, Hilden, Germany) or Myeloid Solutions panel (MYS) (SOPHiA GENETICS, Saint Sulpice, Switzerland). Other tests include an in-house multiplexed (*FLT3*-ITD and *FLT3*-TKD) DNA fragment analysis PCR by capillary electrophoresis using the Applied Biosystem 3500 Series Genetic Analyzer (Thermo Fisher Scientific, Waltham, MA, USA); High-Resolution Melt (HRM) analysis using custom M13-*FLT3*-TKD Forward/Reverse primers by Invitrogen (Thermo Fisher Scientific, Waltham, MA, USA) and performed on the QuantStudio 5 real-time PCR System (Thermo Fisher Scientific, MA, USA). Patients with *FLT3*-TKD variants by HRM analysis proceeded to Sanger sequencing with M13 bacteriophage universal primers (Thermo Fisher Scientific, Waltham, MA, USA) and analyzed by Applied Biosystem 3730XL Series Genetic Analyzer (Thermo Fisher Scientific, Waltham, MA, USA), and trace sequence results were analyzed by Finch TV (Geospiza, Seattle, WA, USA).

Sensitivity measures the proportion of actual positives that are correctly identified by the test (Sensitivity = True Positives/(True Positives + False Negatives)). Specificity measures the proportion of actual negatives that are correctly identified by the test (Specificity = True Negatives/(True Negatives + False Positives)). A specificity and sensitivity of at least 95% is considered acceptable.

Limit of detection (LoD): LoD is defined as the lowest concentration/analyte detected with at least 95% confidence (i.e., <5% false negative results). The LoD was tested using 132 ng of DNA input per well to achieve a theoretical LoD of 0.01% [11]. The amplitude threshold for positive droplets was established for each assay individually by testing the sensitivity and specificity at a range of thresholds (fluorescence value of 2500–6000). Each sample was tested in triplicate, and three positive droplets over the three replicates were required for a positive call. A dilutional study was performed to test assay sensitivity at VAF of 1%. 0.1%, 0.01% and 0.005%. As up to 16 replicates were performed in the dilution studies, the positivity rate is calculated based on the total number of triplicate combinations of all replicates.

Limit of Blank (LoB): The LoB is based on the number of false positive droplets when wild-type samples are tested. Each assay was used on DNA extracted from peripheral blood collected from six healthy donor samples, tested in duplicates with DNA input of 132 ng, and sample amplitude thresholds that were the same as those used for LoD studies.

Linearity: Linearity is the assessment of the ability of a diagnostic test to provide results proportional to the concentration of the measurand. Linearity was assessed via dilutions studied over at least four clinically relevant concentrations (baseline, 1%, 0.1% and 0.01%). The results were plotted as expected VAF versus actual VAF using the statistical package R version 4.3.1 (Vienna, Austria) and expressed as a linear model *y = a + bx* where a = y-intercept of the best line fit, and b = the slope of the best line fit. The coefficient of determination (*R*^2^) is also reviewed to assess non-linearity.

Robustness: Robustness is related to the effect of minor changes in experimental conditions. It was specifically tested for the effect of varying annealing temperature and change in digestion enzyme (*Hae*III to *Mse*I). A standard annealing temperature gradient protocol set by the Bio-Rad C1000 Touch Thermal Cycle (53–58 °C) was used. The effect of these changes on results was analyzed by visualizing the fluorescence plot and calculating the coefficient of variation between the replicates tested with changed conditions.

Precision: Precision is defined by the measure of closeness between independent test results obtained under stipulated conditions, which was reported as repeatability and reproducibility. Repeatability is the precision estimate obtained from tests performed during a short interval by one operator under conditions as similar as possible. In this study, intra-run replicates were performed (up to 12–16) to evaluate repeatability with results expressed as calculated mean, standard deviation, and coefficient of variation (CV = standard deviation/mean). Reproducibility involves measurements made under more variable conditions, and in this validation study, testing of the same samples (over 2–4 times) over a 1-year period by three different operators was incorporated into reproducibility studies. Inter-laboratory reproducibility is also considered as a measurement of reproducibility; however, as no other laboratories performed the same assay, this could not be assessed in our validation study.

Cross-Reactivity: Cross-reaction occurs when the ddPCR assay detects an alternate variant from the specifically targeted variant. This may occur in the presence of variants with a one-base mismatch to the targeted variant. In ddPCR, this may manifest as a fluorescence cluster that is distinct from wild-type and true-positive samples. Cross-reactivity was assessed visually by testing samples with known D835E, D835Y, D835H, and D835V variants with each of the specific *FLT3*-TKD assays targeting D835E, D835Y, D835H, and D835V.

Clinical Utility: A diagnostic test’s clinical utility refers to its applicability in affecting clinical decision making and improving patient outcomes. To assess the application of this ddPCR assay in the MRD setting, DNA samples from patients with a new diagnosis of AML were assessed using this assay. The results from this clinical utility study are exploratory, as there are no current guidelines on the actionability of *FLT3*-TKD results in the MRD setting.

Data visualization: The data plots in Figure 1 and Figure 3 were generated using R 4.3.1 (Vienna, Austria) and the ggplot2 package.

## Figures and Tables

**Table 1:** The accuracy of *FLT3*-TKD D835Y, D835V, D835H, and D835E assays.

Sample Code	HRM	Sanger Sequencing	ddPCR Allelic Frequency	NGS VAF or AR by CE	Concordance
E1	Detected	Not detected	4.97%	4.8%	Yes
H1	Detected	Not detected	3.77%	5.7% *	Yes
H2	Detected	D835H	41.70%	n/p	Yes
H3	Detected	D835H	24.75%	25.4% *	Yes
H4	Detected	D835H	6.77%	6%	Yes
H5	Detected	D835H	25.30%	n/p	Yes
V1	Detected	D835V	20.10%	n/p	Yes
V2	Detected	D835V	26.90%	n/p	Yes
V3	Detected	D835V	42.30%	n/p	Yes
V4	Detected	Not detected	6.29%	n/p	Yes
Y1	Detected	Not detected	5.96%	5%	Yes
Y2	Detected	D835Y	10%	10%	Yes
Y3	Detected	D835Y	21.30%	n/p	Yes
Y4	Detected	D835Y	45.60%	42%	Yes
Y5	Detected	D835Y	18.20%	13%	Yes
Y6	Detected	D835Y	25.54%	42%	Yes
Y7	Detected	D835Y	6.98%	7%	Yes
Y8	Detected	D835Y	38.30%	n/a	Yes
Y9	Not detected	Not detected	1%	1%	Yes
Y10	Detected	D835Y	4.42%	4%	Yes
Y11	Detected	D835Y	26.58%	25.20%	Yes
Y12	Detected	D835Y	3.59%	n/p	Yes
WT1	Not detected	n/p	0.00%	n/p	Yes
WT2	Not detected	n/p	0.00%	n/p	Yes
WT3	Not detected	n/p	0.00%	n/p	Yes
WT4	Not detected	n/p	0.00%	n/p	Yes
WT5	Not detected	n/p	0.00%	n/p	Yes
WT6	Not detected	n/p	0.00%	n/p	Yes

* Allelic ratio was converted to allelic frequency. n/p: Not performed.

**Table 2:** Limit of detection of FLT3-TKD D835Y, D835V, D835H, and D835E assays.

Allelic Frequency	D835Y			D835V			D835H			D835E		
	Replicates	Triplicate Combinations *	Positive Combinations (%)	Replicates	Triplicate Combinations *	Positive Combinations (%)	Replicates	Triplicate Combinations *	Positive Combinations (%)	Replicates	Triplicate Combinations *	Positive Combinations (%)
1%	2	NA	NA	2	NA	NA	4	4	4 (100%)	4	4	4 (100%)
0.1%	7	35	35 (100%)	4	4	4 (100%)	4	4	4 (100%)	4	4	4 (100%)
0.01%	16	560	560 (100%)	12	220	215 (97.5%)	10	120	120 (100%)	12	220	220 (100%)
0.005%	4	4	4 (100%)	NA	NA	NA	4	4	2 (50%)	4	4	4 (100%)

* The total triplicate combination is calculated using the formula n!/(k!(n − k)!), where n is the total number of replicates, and k is 3 (the number of replicates used in each measurable residual disease assay).

**Table 3:** Intra-run variability of FLT3-TKD D835Y, D835V, D835H, and D835E assays.

Allelic Frequency	D835Y		D835V		D835H		D835E	
	Mean ± SD (*n*)	CV%	Mean ± SD (*n*)	CV%	Mean ± SD (*n*)	CV%	Mean ± SD (*n*)	CV%
1%	0.99 ± 0.65 (7)	6.6	0.82 ± 0.009 (2)	1.1	1.46 ± 0.063 (4)	4.3	2.23 ± 0.142 (4)	6.4
0.1%	0.13 ± 0.029 (7)	21.6	0.09 ± 0.026 (4)	27.7	0.14 ± 0.013 (4)	9.4	0.29 ± 0.041 (4)	13.9
0.01%	0.02 ± 0.008 (16)	41.1	0.02 ± 0.008 (12)	54.2	0.018 ± 0.002 (10)	12.6	0.029 ± 0.011 (16)	38.9

SD = standard deviation; CV = coefficient of variation.

**Table 4:** Inter-run variability of FLT3-TKD D835Y, D835V, D835H, and D835E assays.

D835 Variant	Replicates	Mean Allelic Frequency	SD	CV%
D835Y	4	25.53	0.26	1%
D835V	3	42.43	0.26	0.6%
D835H	2	25.16	0.14	0.6%
D835E	2	4.93	0.03	0.7%

SD = standard deviation; CV = coefficient of variation.

## Data Availability

Original data related to this study can be requested from the corresponding author and will be provided if within the scope of ethics approval.

## Supplementary Materials

The following supporting information can be downloaded at https://www.mdpi.com/article/10.3390/ijms25115771/s1.
