# Supplementary material for: FLT3-TKD Measurable Residual Disease Detection Using Droplet Digital PCR and Clinical Applications in Acute Myeloid Leukemia
Source: Int J Mol Sci. 2024 May 26;25(11):5771. doi: 10.3390/ijms25115771 (PMC11171966; doi:10.3390/ijms25115771)
Supplement: Supplementary file 1 [file ijms-25-05771-s001.zip › ijms-2963583-supplementary.pdf]

Supplementary Table S1. Clonal trajectory of FLT3-TKD measurable residual disease in relation to other disease specific markers.

Patient ID	Days since treatment initiation	FLT3-TKD D835E %	KMT2Ar/ABL1 NCN%	BM Blasts %
E1	-5	4.97	100	95
	28	0	0.18	1
	68	0	0.01	2
	236	0	0.03	2

Patient ID	Days since treatment initiation	FLT3-TKD D835Y %	NPM1/ABL1 NCN%	BM Blasts %
Y6	1	44.98	100	50
	70	0	Detected <Below LOQ	3
	148	0	0	1
	253	0	0	1
	406	0	0	1

Patient ID	Days since treatment initiation	FLT3-TKD D835Y %	KMT2A	BM Blasts %
Y8	-10	25.49		91
	18	NA		1
	61	NA		1
	131	0	MRD not performed	1
	200	0		1
	269	0		1
	886	0		2

Patient ID	Days since treatment initiation	FLT3-TKD D835Y %	NPM1/ABL1 NCN%	BM Blasts %
Y9	0	7.08	100	45
	115	0	0.59	1
	158	0	0.04	2
	191	0	Detected <Below LOQ	NA
	218	0	Detected <Below LOQ	NA

Patient ID	Days since treatment initiation	FLT3-TKD D835Y %	FLT3-ITD %	BM Blasts %
Y11	-3	4.47	8.63	77
	21	0.38	6.53	44
	57	0.54	11.14	9
	106	0.54	32.95	55
	134	0.3	15.34	4
	248	NA	NA	1
	345	NA	NA	1
	505	0	49.57	79

Patient ID	Days	FLT3-TKD D835Y %		BM Blasts %
Y12	0	4.47		85

	33	0.38		80	
	55	0.54		50	
**Patient ID**	**Days**	**FLT3-TKD D835Y %**	**FLT3-ITD %**	**CBFB::MYH11/ABL1 NCN%**	**BM Blasts %**
Y14	-6	3.59	2.1	100	80
	29	0	NA	0	1
	56	0	NA	0	3
	97	0	0	0	1

LOQ: Limit of quantification. NCN: Normalized copy number, NCN% is calculated by determining the ratio of oncogenic transcript copy number to *ABL1* copy number divided by the ratio at diagnosis times by 100.

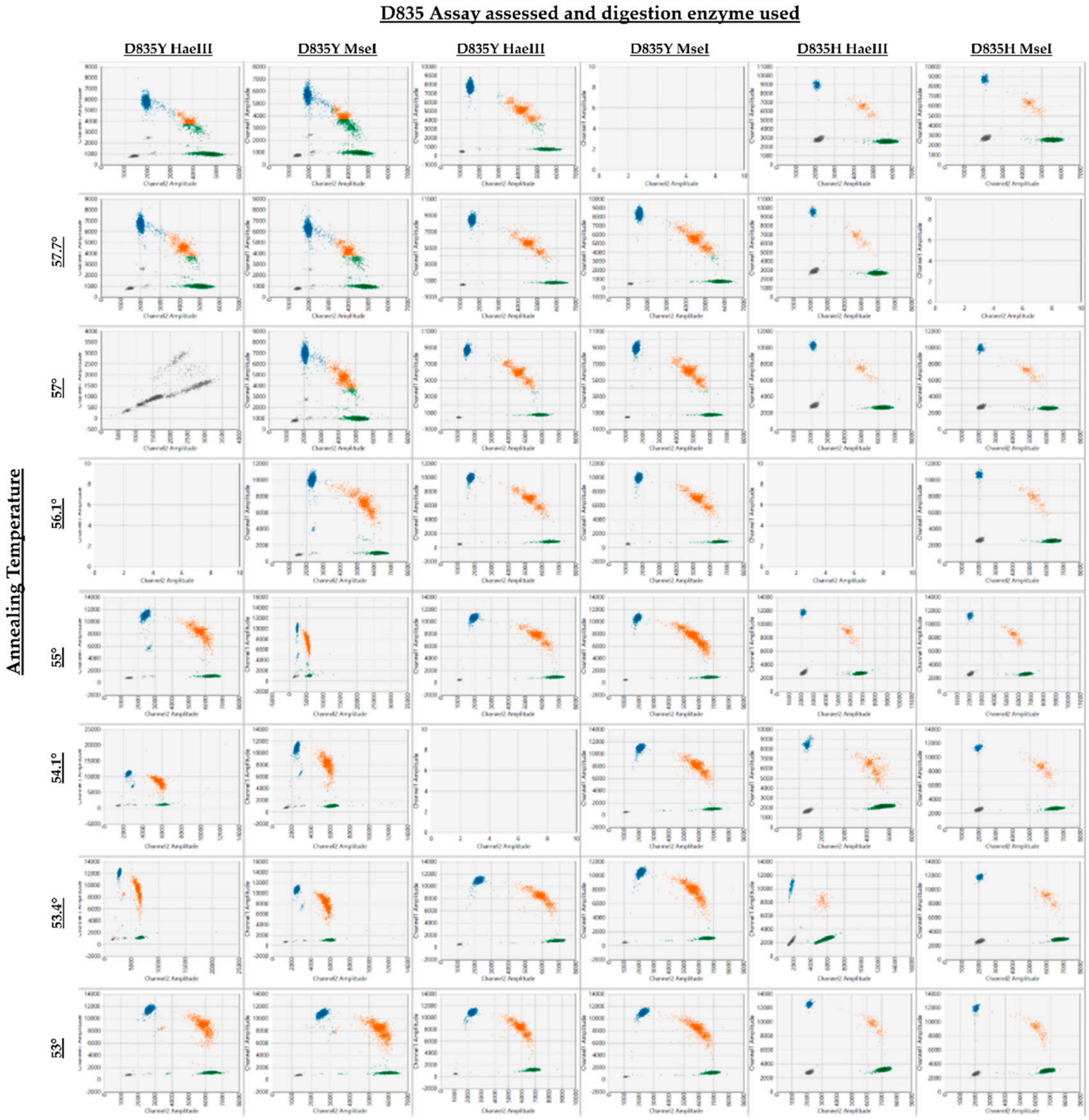

Supplementary Figure S1. Assay robustness assessment, evaluating the effect of changing annealing temperature and digestion enzyme on droplet generation cluster quality. Five samples were excluded due to poor/aberrant droplet generation.
